# Elucidating the Synergistic Effect of Multiple Chinese Herbal Prescriptions in the Treatment of Post-stroke Neurological Damage

**DOI:** 10.3389/fphar.2022.784242

**Published:** 2022-03-09

**Authors:** Anqi Xu, Zhuo-Hua Wen, Shi-Xing Su, Yu-Peng Chen, Wen-Chao Liu, Shen-Quan Guo, Xi-Feng Li, Xin Zhang, Ran Li, Ning-Bo Xu, Ke-Xin Wang, Wen-Xing Li, Dao-Gang Guan, Chuan-Zhi Duan

**Affiliations:** ^1^ Guangdong Provincial Key Laboratory on Brain Function Repair and Regeneration, Department of Neurosurgery, National Key Clinical Specialty/Engineering Technology Research Center of Education Ministry of China, Neurosurgery Institute, Zhujiang Hospital, Southern Medical University, Guangzhou, China; ^2^ Department of Biochemistry and Molecular Biology, School of Basic Medical Sciences, Southern Medical University, Guangzhou, China; ^3^ Guangdong Provincial Key Laboratory of Single Cell Technology and Application, Southern Medical University, Guangzhou, China

**Keywords:** stroke, traditional Chinese medicine, compound-target network, synergy, neuroinflammation

## Abstract

**Background:** Traditional Chinese medicine (TCM) has been widely used in the treatment of human diseases. However, the synergistic effects of multiple TCM prescriptions in the treatment of stroke have not been thoroughly studied.

**Objective of the study:** This study aimed to reveal the mechanisms underlying the synergistic effects of these TCM prescriptions in stroke treatment and identify the active compounds.

**Methods:** Herbs and compounds in the Di-Tan Decoction (DTD), Xue-Fu Zhu-Yu Decoction (XFZYD), and Xiao-Xu-Ming Decoction (XXMD) were acquired from the TCMSP database. SEA, HitPick, and TargetNet web servers were used for target prediction. The compound-target (C-T) networks of three prescriptions were constructed and then filtered using the collaborative filtering algorithm. We combined KEGG enrichment analysis, molecular docking, and network analysis approaches to identify active compounds, followed by verification of these compounds with an oxygen-glucose deprivation and reoxygenation (OGD/R) model.

**Results:** The filtered DTD network contained 39 compounds and 534 targets, the filtered XFZYD network contained 40 compounds and 508 targets, and the filtered XXMD network contained 55 compounds and 599 targets. The filtered C-T networks retained approximately 80% of the biological functions of the original networks. Based on the enriched pathways, molecular docking, and network analysis results, we constructed a complex network containing 3 prescriptions, 14 botanical drugs, 26 compounds, 13 targets, and 5 pathways. By calculating the synergy score, we identified the top 5 candidate compounds. The experimental results showed that quercetin, baicalin, and ginsenoside Rg1 independently and synergistically increased cell viability.

**Conclusion:** By integrating pharmacological and chemoinformatic approaches, our study provides a new method for identifying the effective synergistic compounds of TCM prescriptions. The filtered compounds and their synergistic effects on stroke require further research.

## Introduction

Stroke is the second leading cause of death and disability worldwide and can be categorized into two subtypes: ischemic stroke (cerebral infarction) and hemorrhagic stroke (intracerebral hemorrhage and subarachnoid hemorrhage) (Collaborators, 2019). Within a very short time, approximately several hours after stroke, neurological damage needs to be minimized by removing blockages or hemorrhages with surgery and medications. After the most effective therapies are administered in the short term, there is still a lack of specific medicines for stroke. In the clinic, symptomatic and supportive medications, including neurotrophic medicines (e.g., edaravone) (Feng et al., 2011; Yang et al., 2011) and cytidine (Marti-Carvajal et al., 2020)); antiplatelet medicines (e.g., aspirin and clopidogrel (Johnston et al., 2018)); and anti-vasospasm medicines (e.g., nimodipine) (Carlson et al., 2020), are mainly used in stroke treatment. However, currently available therapies, especially medications, are still limited. Therefore, there is an urgent need for more effective treatment of post-stroke neurological damage.

Traditional Chinese medicine (TCM) has a long history of treating post-stroke neurological damage (Gong and Sucher, 1999). Di-Tan Decoction (DTD), Xue-Fu Zhu-Yu Decoction (XFZYD), and Xiao-Xu-Ming Decoction (XXMD) are three widely used classical prescriptions, and they cover the three most common syndromes in post-stroke patients complementarily, including phlegm obstruction, blood stasis, and Qi deficiency (Fu et al., 2013; Shi et al., 2013; Shen et al., 2015; Yang et al., 2020; Kwon et al., 2021). Previous studies have shown that DTD could promote metabolism and improve blood circulation by resolving phlegm (lei et al., 2020; Kwon et al., 2021), alleviating syncope, and improving movement and speech after stroke (Yan et al., 2017; Xue, 2020). XFZYD was reportedly the most representative prescription for removing blood stasis and promoting blood circulation in TCM prescriptions (Yi and Hongyan, 2018; Shao et al., 2020). It contained a variety of herbs with significant anticoagulation effects, which were important for improving the local blood supply and controlling the recurrence of thrombosis (Lee JJ. et al., 2011; Yi and Hongyan, 2018; Hongqin et al., 2019). XXMD reportedly promoted Qi to activate blood in TCM experience (Fu et al., 2013). It was usually used to treat systemic symptoms after stroke, including mouth and eyes askew, slurred speech, body paralysis, dizziness, phlegm accumulation, drooling, etc. (Fu et al., 2013; Juan et al., 2021).

To explore the efficacy and safety of DTD, XFZYD, and XXMD in stroke treatment with modern clinical studies, we searched both Chinese and English literature databases for RCTs using the three prescriptions. In the Chinese database, DTD, XFZYD, and XXMD respectively have 53, 79, and 25 stroke-related RCT studies. Among which, 37, 62, and 21 are ischemic stroke-related RCTs, respectively, and 16, 17, and 4 are hemorrhagic stroke-related RCTs, respectively. For example, Yan et al. reported that DTD significantly promoted neurological recovery of 32 ischemic stroke patients compared with the control group (Yan et al., 2017). Hong et al. reported that XFZYD improved muscle strength recovery and decreased blood viscosity of 100 patients after ischemic stroke (Hongqin et al., 2019). Juan et al. reported that the application of XXMD based on conventional treatment reduced NIHSS score and the levels of TNF-α, IL-6, and hs-CRP in 43 acute ischemic stroke patients (Juan et al., 2021). We believe that these Chinese RCTs could provide positive evidence on efficacy and safety, which correspond to the previous meta-analysis results (Fu et al., 2013; Shao et al., 2020; Kwon et al., 2021). In the English database, we only collected 2 RCTs related to DTD and XFZYD with other diseases, which showed the efficacy and safety of DTD in treating Alzheimer’s disease and XFZYD in treating unstable angina (Chu et al., 2010; Chua et al., 2015).

In addition, several previous pharmacological studies showed that DTD, XFZYD, and XXMD had neuroprotective effects and therapeutic potential *in vitro* and *in vivo*. DTD reportedly decreased inflammation and oxidative stress by inhibiting ERK, Nrf2/HO-1, and NF−kappa B signaling pathways (Kwon et al., 2021). XFZYD could inhibit adenosine diphosphate-induced platelet activation through blocking exposure of the glycoprotein IIb/IIIa complex (Li et al., 1999), and XFZYD mediated neuroprotection via inhibition of HIF-1α and TNF-α, followed by inhibition of inflammatory responses and apoptosis (Lee JJ. et al., 2011). XXMD was revealed to exert neuroprotective effects by downregulating the expression levels of LC3, Beclin1, Lamp1, and mitochondrial p62, thereby inhibiting mitochondrial autophagy (Lan et al., 2018). Although there are some similarities between mice and humans in cerebral vascular anatomy and post-stroke pathophysiology, there are also some important differences. For example, a higher ratio of gray matter to white matter in the brains of mice leads to more intense neurological function after stroke, as the rats’ neurons tolerate hypoxia longer and the astrocytes resist oxidative stress better than humans (Casals et al., 2011; Krafft et al., 2012). Moreover, rodents demonstrated better neuroprotection and a smaller risk of bleeding at thrombin blockade when compared with human coagulation function. Thus, some drugs have been shown to work well in stroke rats, but the results have limited application in treating humans (Hermann et al., 2019).

**FIGURE 1:**
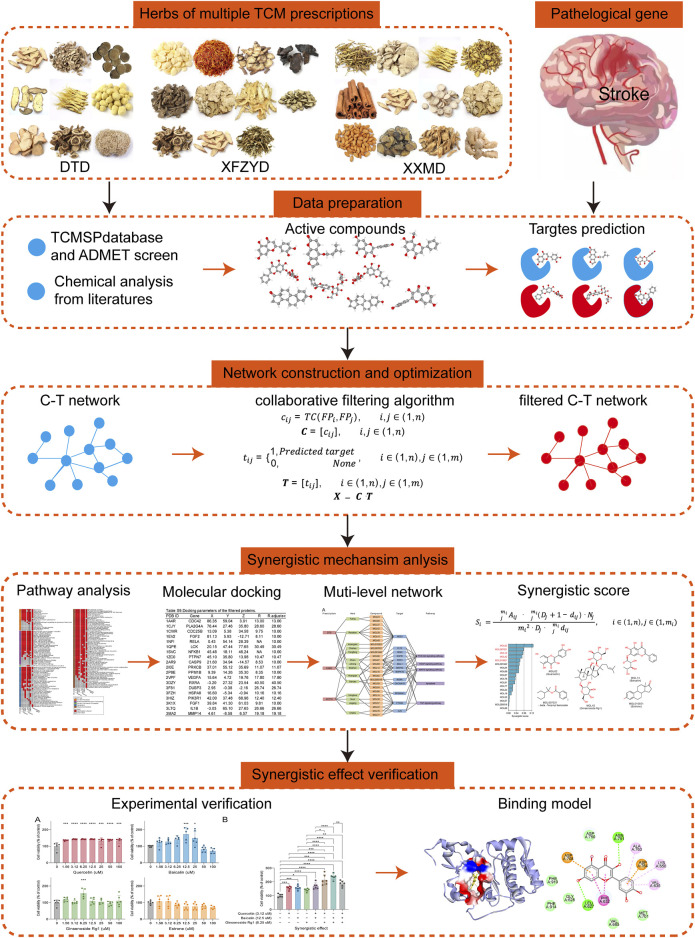
The workflow of the analysis for this study.

These prescriptions are multi-component and multi-targeted prescriptions, and they formed complex compound-target (C-T) networks. As is widely known, Network pharmacology is a commonly used approach for revealing the mechanisms of TCM based on the C-T network (Jiang, 2005; Hopkins, 2008; Lee et al., 2019; Yang L. et al., 2021). It is a common phenomenon that multiple prescriptions like DTD, XFZYD, and XXMD are used for reference or in conjunction with each other in TCM clinical practice. However, the synergetic relationship in C-T networks of multiple prescriptions remains unclear. An important reason is the lack of a suitable model based on network pharmacology. In this study, we propose a new model via integrating network pharmacology and chemoinformatic approaches to elucidate the synergistic effects of DTD, XFZYD, and XXMD in the treatment of post-stroke neurological damage. The workflow our our study was showed with Figure 1.

## Materials and Methods

### Collection of Traditional Chinese medicine Prescriptions and Components

Botanical drugs with origin and dosage in DTD, XFZYD, and XXMD were retrieved from the Chinese pharmacopeia 2020 (National Pharmacopoeia Commission, 2020) and the Traditional Chinese Medicine Systems Pharmacology (TCMSP) database (https://tcmsp-e.com/) (Ru et al., 2014), and then proofread the Latin names of herbs by The Plant List database (https://www.theplantlist.org) (List, 2013). The name, origin, and dosage of botanical drugs in these prescriptions are listed in Table 1. Information concerning the chemical components of these botanical drugs was obtained from the TCMSP database and a literature search. Chemical structures were prepared and converted into canonical SMILES using OpenBabel Toolkit (version 3.1.1) (O’Boyle et al., 2008).

**TABLE 1:** The name, origin, and dose of these botanical drugs in the three prescriptions.

Chinese name	Latin name	Origin	Dose (g)
Ditan Decoction (DTD)			
Banxia	Pinellia ternata (Thunb.) Makino (Araceae; root of Pinellia ternata)	Hubei, China	12.5
Fuling	Poria cocos (Schw.)Wolf (Polyporaceae; Wolfiporia)	Yunnan, China	10
Gancao	Glycyrrhiza uralensis Fisch (Leguminosae; licorice root)	Inner Mongolia, China	2.5
Juhong	Citrus reticulata Blanco (Rutaceae; Red tangerine peel)	Guangdong, China	7.5
Nanxing	Arisaema heterophyllum Blume (Araceae; Reddish jackinthepulpit root)	Hubei, China	12.5
Renshen	Panax ginseng C.A.Mey. (Araliaceae; Ginseng root)	Jilin, China	5
Shichangpu	Acorus calamus var. angustatus Besser (Acoraceae; Grassleaved sweetflag)	Sichuan, China	5
Zhishi	Citrus × aurantium L. (Rutaceae; immature bitter orange)	Sichuan, China	10
Zhuru	Bambusa tuldoides Munro (Poaceae; Bamboo shavings)	NA	3.5
Xuefu Zhuyu Decoction (XFZYD)			
Chaihu	Bupleurum chinense DC. (Apiaceae; Chinese thorowax root)	Anhui, China	3
Chishao	Paeonia obovata Maxim. (Paeoniaceae; Red peony root)	NA	6
Chuanxiong	Cnidium officinale Makino (Apiaceae; cnidium officinale)	Sichuan, China	4.5
Danggui	Angelica sinensis (Oliv.) Diels (Apiaceae; Radix angelicae sinensisradix)	Gansu, China	9
Dihuang	Rehmannia glutinosa (Gaertn.) DC. (Plantaginaceae; rehmannia root)	Henan, China	9
Gancao	Glycyrrhiza uralensis Fisch (Leguminosae; licorice root)	Inner Mongolia, China	6
Honghua	Carthamus tinctorius L. (Compositae; safflower flower)	Henan, China	9
Jiegeng	Platycodon grandiflorus (Jacq.) A.DC. (Campanulaceae; balloonflower root)	Jilin, China	4.5
Niuxi	Achyranthes bidentata Blume (Amaranthaceae; Twotooth achyranthes root)	Henan, China	9
Taoren	Prunus persica (L.) Batsch (Rosaceae; Peach kernel)	Hubei, China	12
Zhike	Citrus × aurantium L. (Rutaceae; peel of trifoliate orange)	Jiangxi, China	6
Xiaoxuming Decoction (XXMD)			
Baishao	Paeonia lactiflora Pall. (Paeoniaceae; white peony root)	Zhejiang, China	9
Chuanxiong	Cnidium officinale Makino (Apiaceae; cnidium officinale)	Sichuan, China	3
Fangfeng	Saposhnikovia divaricata (Turcz.) Schischk. (Apiaceae; divaricate saposhnikovia root)	Heilongjiang, China	6
Fangji	Stephania tetrandra S.Moore (Menispermaceae; fourstamen stephania root)	Hubei, China	6
Fuzi	Aconitum carmichaeli Debx. (Ranunculaceae; Prepared common monkshood daughter root)	Henan, China	3
Gancao	Glycyrrhiza uralensis Fisch (Leguminosae; licorice root)	Inner Mongolia, China	3
Huangqin	Scutellaria baicalensis Georg (Lamiaceae; scutlaria baicalensis root)	Inner Mongolia, China	6
Kuxingren	Semen Armeniacae Amarum. (Rosaceae; Bitter Apricot Seed)	Inner Mongolia, China	9
Mahuang	Ephedra sinica Stapf (Ephedraceae; Chinese ephedra)	Inner Mongolia, China	3
Renshen	Panax ginseng C.A.Mey. (Araliaceae; Ginseng root)	Jilin, China	3
Rougui	Cinnamomum cassia (L.) J.Presl (Lauraceae; cinnamon)	Guangxi, China	3
Shengjiang	Zingiber officinale Roscoe (Zingiberaceae; fresh ginger)	NA	9

### Collection of Stroke Pathological Gene

Genes related to stroke pathology were collected from multiple public databases. We used “stroke” as the keyword to search for genes in the Clarivate Analytics Integrity database (https://integrity.clarivate.com/integrity/xmlxsl/), therapeutic target database (TTD, http://db.idrblab.net/ttd/) (Wang Y. et al., 2020), DisGeNet database (https://www.disgenet.org/) (Pinero et al., 2020), and GeneCards (https://www.genecards.org/) (Safran et al., 2010). The pathological genes were defined as the intersection of the genes searched by GeneCards and the union set of the genes searched by Integrity, TTD, and DisGeNet.

### Screening of Active Compounds in Traditional Chinese medicine Prescription

The computed physicochemical properties and other parameters of all compounds were obtained from the TCMSP database. According to Lipinski’s rule of five (Pollastri, 2010), active compounds met the following conditions: 1) molecular weight (MW) ≤ 500, 2) hydrogen bond donors (Hdon) ≤ 5, 3) hydrogen bond acceptors (Hacc) ≤ 10, 4) octanol-water partition coefficient (AlogP) ≤ 5, and 5) rotation bond number (RBN) ≤ 10. Furthermore, based on the suggested drug screening criteria listed in the TCMSP, active compounds also met the following additional conditions: 6) oral bioavailability (OB) ≥ 30%, 7) drug likeness (DL) ≥ 0.1, 8) blood–brain barrier (BBB) penetration ≥0.3, 9) Caco-2 permeability (Caco-2) ≥ 0.4, and topological polar surface area (TPSA) ≤ 60 (Wang et al., 2020a; Wang et al., 2020b).

### Compound-Target (C-T) Prediction and Network Construction

Potential targets of the active compounds were predicted by using the similarity ensemble approach (SEA) search server (https://sea.bkslab.org/) (Keiser et al., 2007), HitPickV2 (http://www.hitpickv2.com/) (Hamad et al., 2019), and TargetNet (http://targetnet.scbdd.com) (Yao et al., 2016). The union set of the prediction results of these three tools is defined as the potential targets. The C-T network was constructed and visualized using Cytoscape version 3.8.2 (Kohl et al., 2011) and yFiles layouts (https://www.yworks.com/blog/yfiles-cytoscape-app).

### Optimization of the Compound-Target Network by a Collaborative Filtering Algorithm

The constructed C-T networks were simplified by using the collaborative filtering algorithm. First, the ECFP6 molecular fingerprints for all the compounds were calculated by using the “rcdk” package (Voicu et al., 2020), and then the Tanimoto coefficient (TC) of all paired compounds was calculated. The following is the formula of the algorithm:

$$c_{ij}=TC\left(FP_{i},FP_{j}\right),\;\;i,j\in \left(1,n\right)$$

$$C=\left[c_{ij}\right],\;\;i,j\in \left(1,n\right)$$

Assume that the total number of compounds is n , and $c_{ij}$ indicates the TC of compound i and compound j . The matrix C indicates the TC matrix of all paired compounds.

$$t_{ij}=\{\begin{array}{c} 1,Predicted\;target\;\\ 0,\;\;\;\;\;\;\;\;\;\;\;\;\;\;\;\;\;\;\;\;\;\;\;\;\;None \end{array},\;\;i\in \left(1,n\right),j\in \left(1,m\right)$$

$$T=\left[t_{ij}\right],\;\;i\in \left(1,n\right),j\in \left(1,m\right)$$

Assume that the total number of targets is m , and $t_{ij}$ indicates whether the target j is the predicted target of compound i . The matrix T indicates the C-T matrix of all filtered compounds and predicted targets in the C-T network.

X=C·T

$$s_{i}=\frac{1}{m}\overset{m}{\underset{j=1}{\sum }}x_{ij},\;\;i\in \left(1,n\right),j\in \left(1,m\right)$$

Matrix X is obtained by multiplying matrix C and T , and $x_{ij}$ is an element in matrix X . The score $s_{i}$ indicates the synergy score of compound i . The top 50% of the compounds and their targets were filtered for further analysis.

### GO and KEGG Enrichment Analysis

GO terms of biological process, cellular component, molecular function, and human gene information were downloaded from the QuickGO database (https://www.ebi.ac.uk/QuickGO/) (Binns et al., 2009). The reference human genes and pathways were obtained from the Kyoto Encyclopedia of Genes and Genomes (KEGG) database (http://www.kegg.jp/) (Ogata et al., 1999). GO terms and KEGG pathways with fewer than 10 genes were removed. The enrichment analysis was performed using the hypergeometric test and the formula shown in a previous report (Liu HY. et al., 2019; Gao et al., 2020). An FDR-corrected *p*-value ≤ 0.05 was considered significantly enriched.

### Molecular Docking

The crystal structures of the filtered targets were downloaded from the Protein Data Bank (PDB) database (https://www.rcsb.org/) (Messenguy, 1987). The filtered molecule SMILES were converted into “. pdb” format by Discovery Studio version 2016. Proteins and compounds in “. pdb” format were converted to “. pdbqt” format for molecular docking using OpenBabel Toolkit. The center position (x, y, z coordinates) and radius of potential binding sites of target proteins were calculated using the From Receptor Cavities tool in Discovery Studio. Molecular docking analysis of the filtered compounds and proteins was performed using AutoDock Vina 1.1.2 (Trott and Olson, 2010) with its default parameters, and the highest affinity (the lowest docking energy) of each docking pair was chosen as the docking result. The compound-target pairs with the lowest docking energy ≤ −8 kcal/mol were screened for further analysis.

### Screen of Synergistic Compounds

The filtered compound-target pairs, prescriptions, botanical drugs, and pathways were used to construct the complex network. We calculated the molecular fingerprint similarity matrix for the compounds in the network. For compound pairs with similarity greater than 0.5, the compound with the largest number of targets was retained. Other compounds with similarity of less than 0.5 were retained. The following formula was used to calculate the synergistic score:

$$S_{i}\;=\frac{\left|\sum_{j}^{m_{i}}A_{ij}\right|\cdot \sum_{j}^{m_{i}}\left(D_{j}+1-d_{ij}\right)\cdot N_{j}}{m_{i}\cdot D_{j}\cdot \sum_{j}^{m_{i}}d_{ij}},\;\;i\in \left(1,n\right),j\in \left(1,m_{i}\right)$$

In the formula, n is the number of screened compounds, and $m_{i}$ is the number of targets for compound i . $\;S_{i}$ is the synergistic score for compound i . $A_{ij}$ is the docking affinity of compound i and target j , and $d_{ij}$ is the distance between compound i and target j . $D_{j}$ is the maximum distance between target j and all compounds. $N_{j}$ is the number of compounds interacting with target j . This formula considers the binding ability of the targets and the control power with other nodes. All compounds were sorted according to the synergistic score from high to low, and the top 5 compounds were selected for experimental verification. The synergy score was calculated based on the compound similarity and the target prediction results. Therefore, the accuracy of the synergy score will be affected by the compound information in the herbs and the accuracy of the target prediction results. Due to incomplete information of compounds in botanical drugs and inaccurate target prediction results, the calculated synergy score may have certain deviations.

### Cell Culture and Oxygen–Glucose Deprivation and Reoxygenation (OGD/R) Model

The HT22 cell line (murine hippocampal cells) was obtained from CHI SCIENTIFIC (Shanghai, China). The cells were maintained in a humidified atmosphere at 37°C, with 5% CO_2_ and a complete culture medium (DMEM supplemented with 10% FBS). Oxygen and glucose deprivation/reoxygenation (OGD/R) is a well-established model to mimic ischemic/reperfusion conditions of cells *in vitro* (Moran et al., 2017). Specifically, the cells were washed three times with PBS, digested by trypsin, and plated in 20,000/200 µL medium per well of a 96-well plate. After 24 h, the cells were changed to low glucose DMEM (1 g/L glucose) and incubated in a hypoxic environment at 1% O_2_, 5% CO_2_, and 37°C for 18 h. After the OGD period, the cells were incubated with a complete culture medium under normoxic conditions for 24 h. In addition, different concentrations of compounds were dissolved in a complete culture medium during the period of reoxygenation. 1/10,000 DMSO in culture medium was used as vehicle control.

### Cell Viability Assay

Cell viability assays were carried out with Cell Counting Kit-8 (RiboBio, China) according to the manufacturer’s instructions. In brief, 10 μL CCK8 solution in 100 μL complete culture medium was added to each well of the 96-well plate and further incubated for 3 h. Then, the absorbance was measured at 450 nm optical density in a microplate reader (KC junior, BioTek, United States). The cell viability was calculated by the mean of the optical density values in 6 repeat wells.

### Materials

Fetal bovine serum (FBS) and Dulbecco’s Modified Eagle’s Medium (DMEM) were purchased from Thermo Fisher Biochemical Products (Beijing) Co., Ltd. Hypoxic bags were purchased from Mitsubishi Gas Chemical Company, Inc. (Japanese). Cell Counting Kit-8 (CCK-8) was purchased from Dojindo Laboratories (Japanese). Quercetin (>98% purity by HPLC), Baicalin (>93.1% purity by HPLC), Ginsenoside Rg1 (>98.6% purity by HPLC), and Estrone (>98% purity by HPLC) were purchased from Jiangsu Yongjian Pharmaceutical Technology Co., Ltd. (Jiangsu, China).

### Statistical Analysis

R statistical software version 4.0.3 was used for statistical analysis of experimental data. Outliers in each repeat that were higher than twofold of the standard deviation were eliminated. Independent sample t-tests were used to compare the individual effects of different concentrations of drugs on cell viability. One-way ANOVA and LSD posthoc tests were used to test the synergistic effects of the active drugs on cell viability. A *p* value less than 0.05 was considered statistically significant.

## Results

### Herbs and Compounds in DTD, XFZYD, and XXMD

We identified 981, 1,688, and 2016 compounds in the DTD, XFZYD, and XXMD, respectively, from the TCMSP database (Supplementary Table S1). According to the rigorous screening criteria, there were 54, 49, and 120 active compounds in DTD, XFZYD, and XXMD, respectively (Supplementary Tables S2–S4). Because there were such few records of the compounds of some herbs were in the TCMSP database, no compounds were found in the Dihuang [Rehmannia glutinosa (Gaertn.) DC. (Plantaginaceae; rehmannia root)], Fuling [Poria cocos (Schw.) Wolf (Polyporaceae; Wolfiporia)], Jiegeng [Platycodon grandiflorus (Jacq.) A. DC. (Campanulaceae; balloonflower root)], Taoren [Prunus persica (L.) Batsch (Rosaceae; Peach kernel)]), or Zhuru [Bambusa tuldoides Munro (Poaceae; Bamboo shavings)] after ADMET screening. Furthermore, we found 33, 45, and 35 herbal compounds in DTD, XFZYD, and XXMD, respectively, through literature search (Supplementary Table S5). The unique compounds screened by ADMET and obtained from the literature search were combined for subsequent analysis.

### Compound-Target Network Construction

The numbers of filtered compounds in DTD, XFZYD, and XXMD were 77, 79, and 109, respectively. After combining these compounds, a total of 171 unique compounds were used for target prediction. Three web servers, SEA, HItPickV2, and TargetNet, were used for compound-target prediction, and the union of prediction results was used for subsequent analysis. The number of predicted targets of these compounds ranged from 23 to 189 (Supplementary Table S6). The above compound-target prediction results were used to construct the C-T network for each prescription.

### Topological Properties of the Original and Filtered Compound-Target Network

**FIGURE 2:**
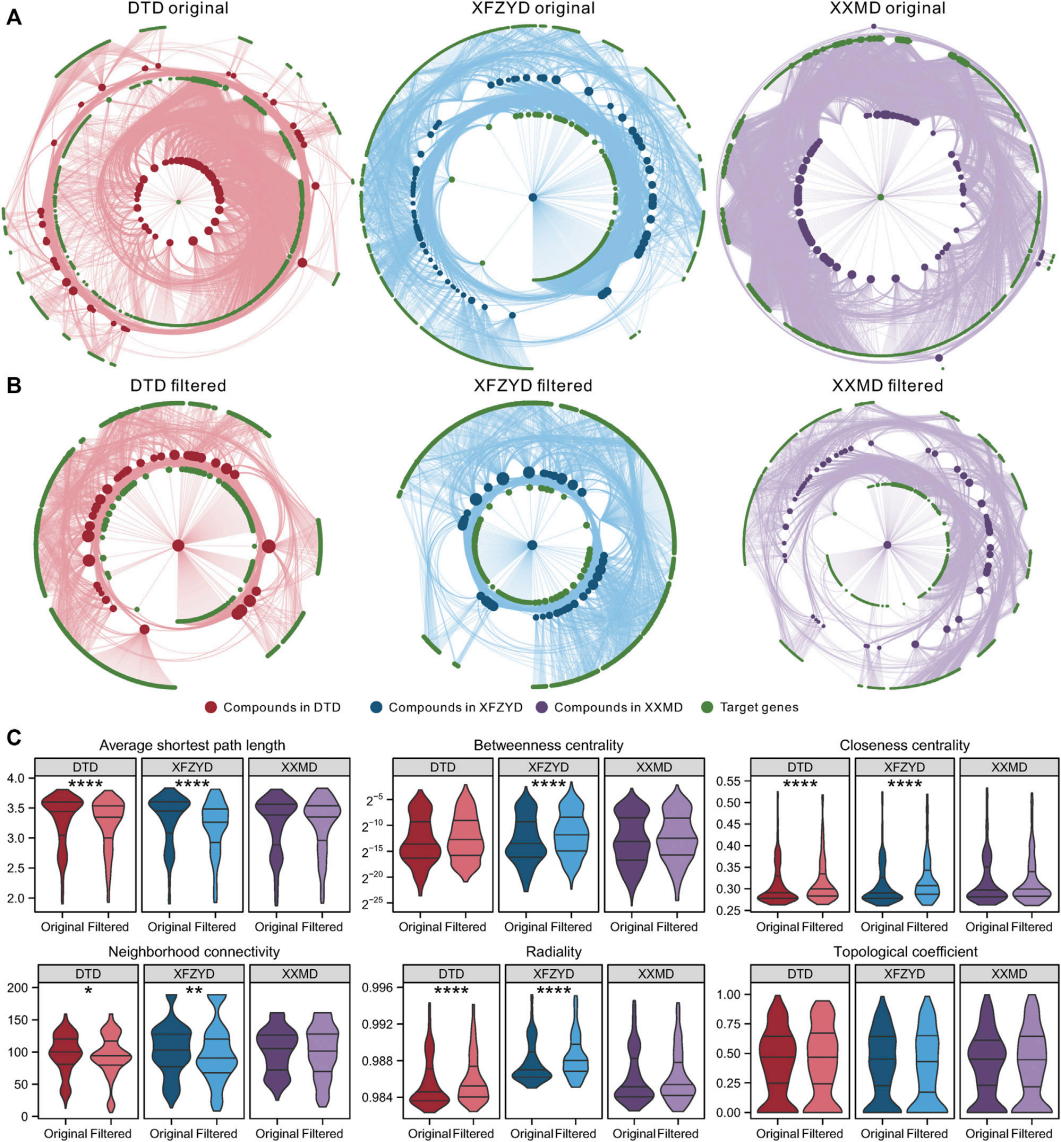
Characteristics and topological properties of the original and filtered compound-target (C-T) networks: The original C-T network of DTD, XFZYD, and XXMD **(A)**. The filtered C-T network of DTD, XFZYD, and XXMD **(B)**. Topological properties of the original and filtered C-T network of DTD, XFZYD, and XXMD **(C)**. Significance: *p < 0.05, **p < 0.01, ***p < 0.001, ****p < 0.0001.

The original C-T network of DTD contains 7,120 compound-target pairs with 77 compounds and 733 targets, the original C-T network of XFZYD contains 7,122 compound-target pairs with 79 compounds and 768 targets, and the original C-T network of XXMD contains 10,372 compound-target pairs with 109 compounds and 755 targets (Figure 2A). All three C-T networks were filtered using the collaborative filtering algorithm. The filtered DTD network contained 3,183 compound-target pairs with 39 compounds and 534 targets, the filtered XFZYD network contained 2,925 compound-target pairs with 40 compounds and 508 targets, and the filtered XXMD network contained 4,837 compound-target pairs with 55 compounds and 599 targets (Figure 2B). There were significant differences in the average shortest path length, closeness centrality, neighborhood connectivity, and radiality between the original and filtered C-T networks of DTD and XFZYD. There was no difference in topological properties between the original and filtered XXMD networks (Figure 2C).

### The Comparison Between the Original and Filtered Compound-Target Networks

**FIGURE 3:**
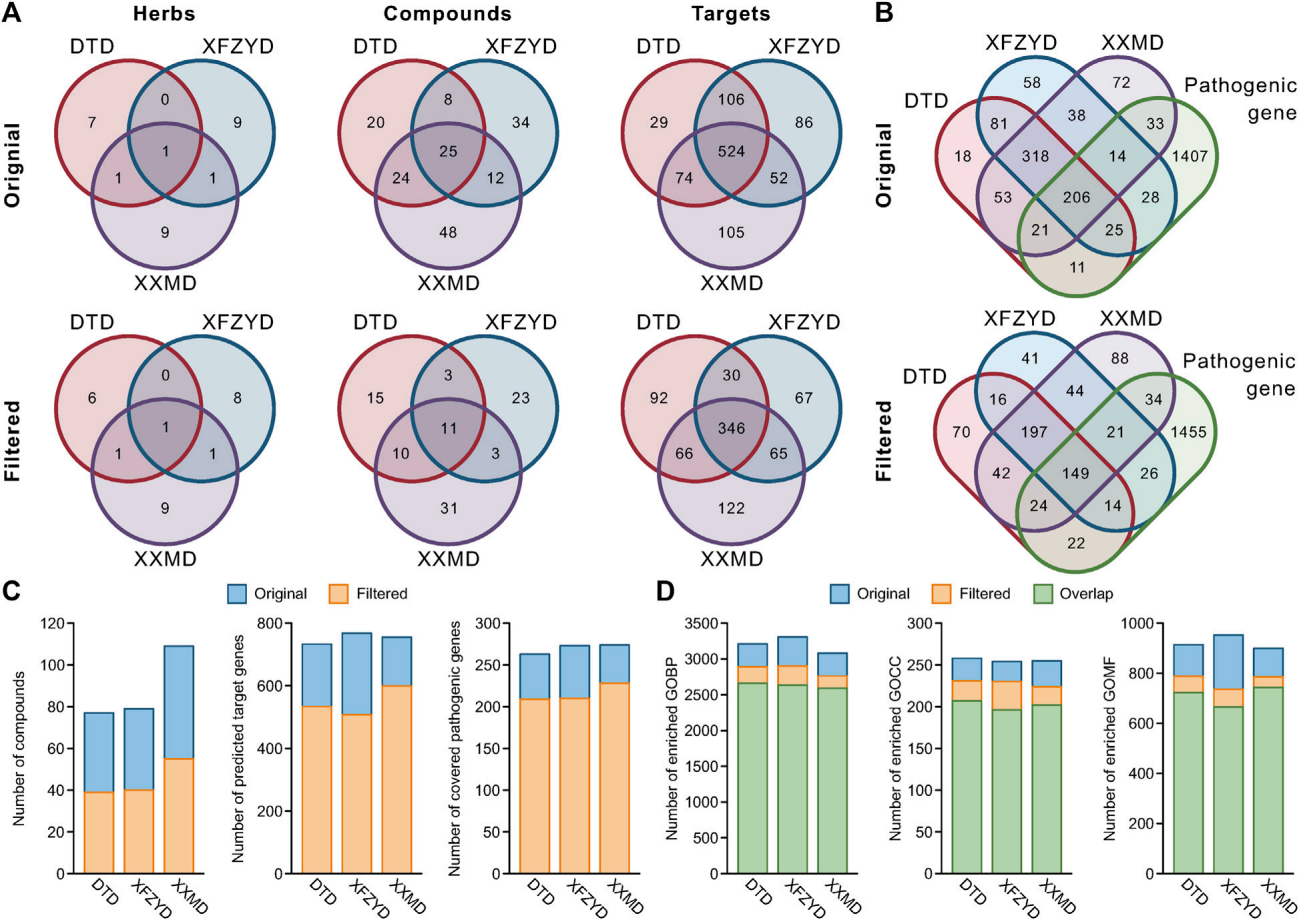
The components and biological functions of the original and filtered compound-target (C-T) networks: Venn diagram of herbs, compounds and predicted targets in the original and filtered C-T networks of DTD, XFZYD, and XXMD **(A)**. Venn diagram of predicted targets and pathological genes in the original and filtered C-T network **(B)**. Number of compounds, predicted target genes, and covered pathological genes in the original and filtered C-T networks of DTD, XFZYD, and XXMD **(C)**. Number of enriched GO biological process (GOBP), GO cellular component (GOCC), and GO molecular function (GOMF) terms in the original and filtered target genes of DTD, XFZYD, and XXMD **(D)**.

Although there were only half of the compounds in the filtered network compared to the original network, most of the herbs (8 out of 9 herbs in DTD, 10 out of 11 herbs in XFZYD, and all herbs in XXMD) and targets (72.9% targets in DTD, 66.1% targets in XFZYD, and 79.3% targets in XXMD) were still retained (Figures 3A,C). Furthermore, the ratio of included pathological genes to the original retained was 79.5, 76.9, and 83.2% (Figures 3B,C). In addition, the proportion of compounds shared by the three prescriptions in the union of all compounds only decreased from 14.6 to 11.5%, and the proportion of targets shared by the three prescriptions in the union of all targets only decreased from 53.7 to 43.9% after filtering (Figure 3A). A total of 1,745 stroke pathological genes were collected in this study (Supplementary Table S7). The intersection of the union targets in three prescriptions and the pathological genes after filtering retained 85.8% (Figure 3B). Finally, comparing the results of GO enrichment, the results indicated that most of the biological functions were maintained in the filtered C-T networks, and that the ratio of overlapped GO biological process to the original were 82.9, 79.6, and 84.1% in DTD, XFZYD, and XXMD, respectively. Additionally, the ratio of overlapped GO cellular components to the original were 80.2, 77.2, and 79.2% in DTD, XFZYD, and XXMD, and the ratio of overlapped GO molecular function to the original were 79.1, 69.9, and 82.6% in DTD, XFZYD, and XXMD, respectively (Figure 3D).

### Key Pathways and Targets Screen Based on Filtered Compound-Target Network

**FIGURE 4:**
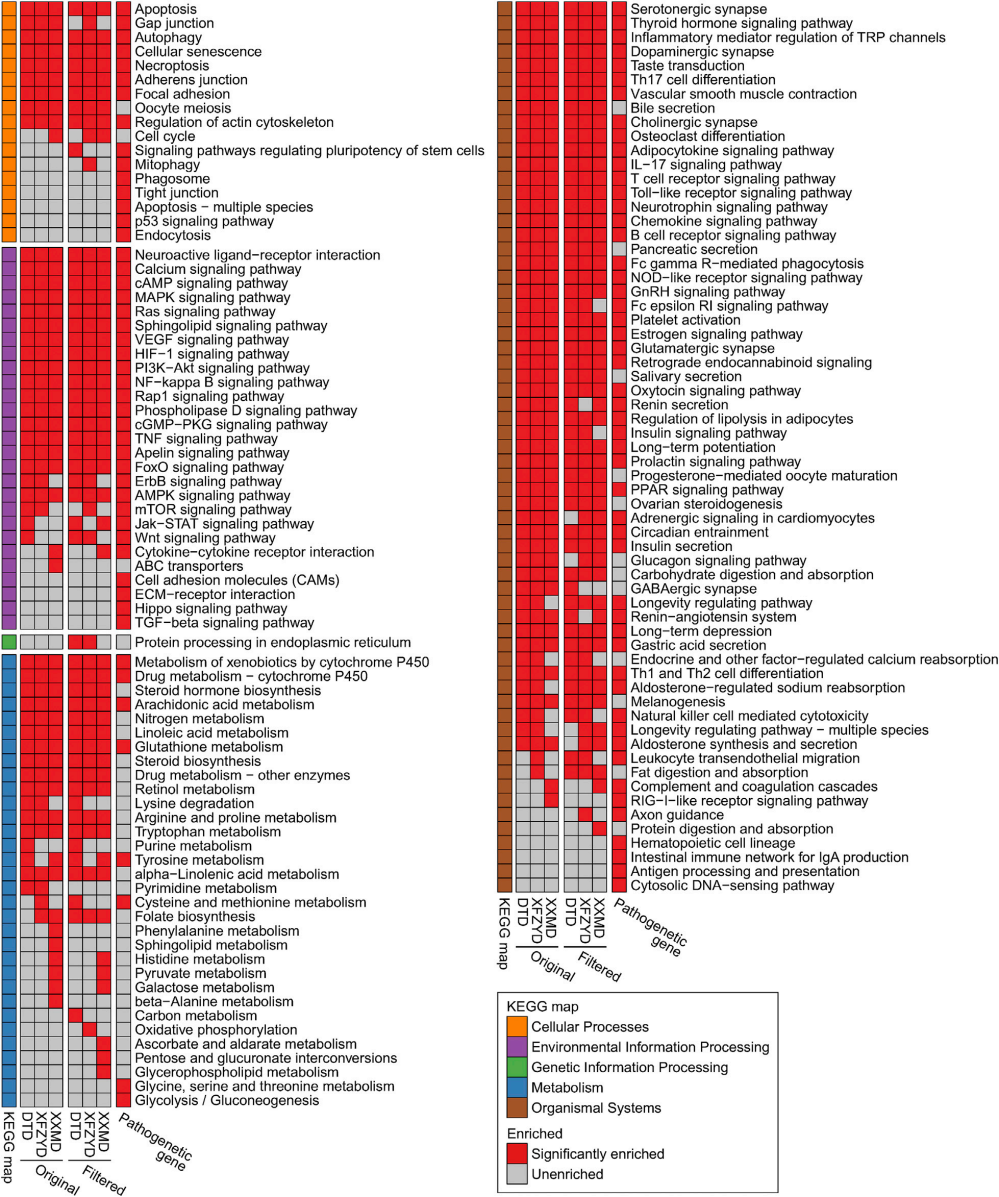
The enriched KEGG pathways of pathological genes and the original and filtered target genes of DTD, XFZYD, and XXMD: The figure shows the union set of the top 100 enriched KEGG pathways (sorted by FDR corrected *p* value) in each group.

**FIGURE 5:**
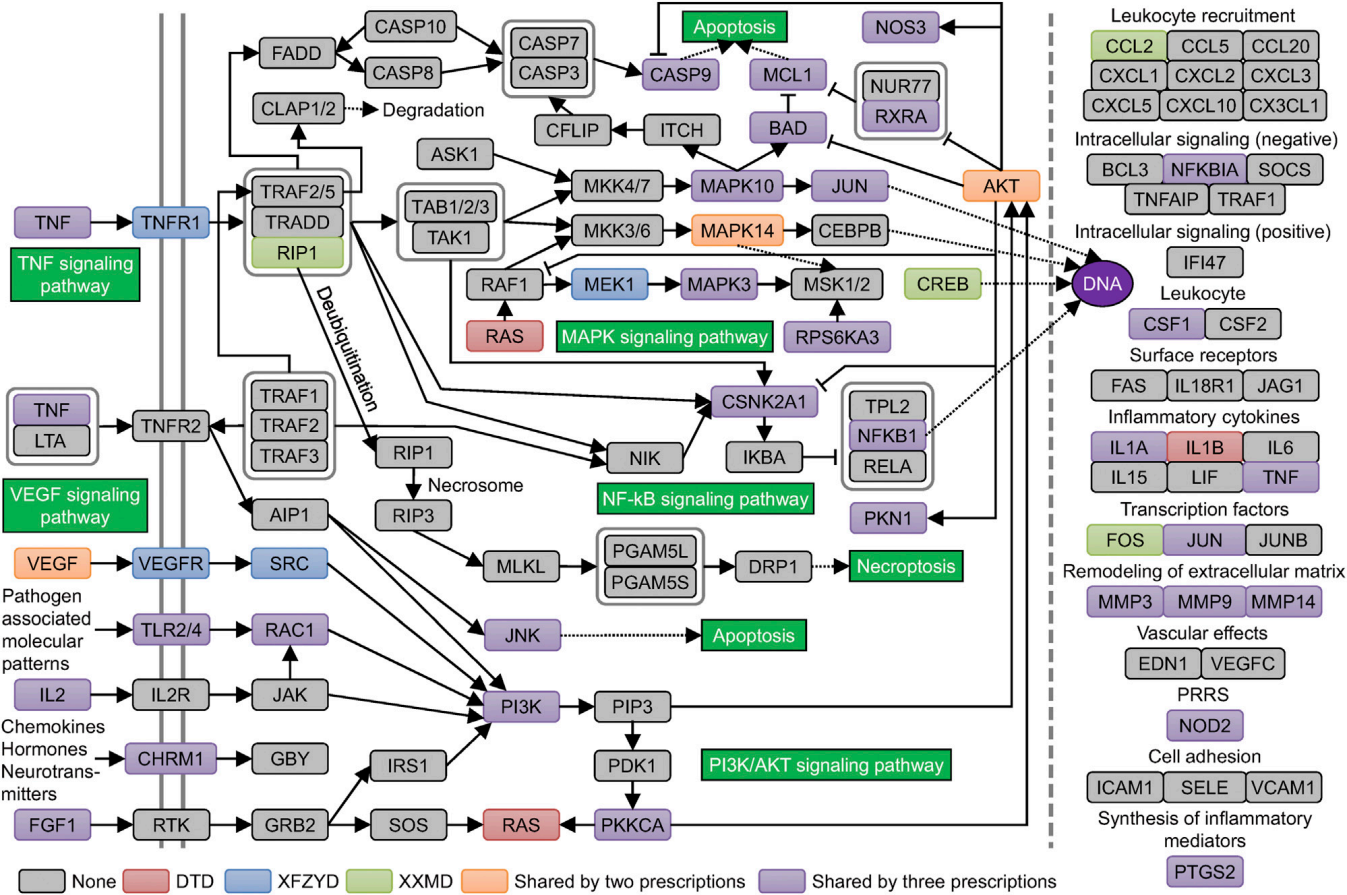
Curated KEGG pathways related to the pathogenesis of stroke: Curated pathways are shown in the green boxes. Genes are shown in colored, rounded rectangles.

KEGG pathway enrichment results showed that the genes related to stroke pathology and the original and filtered target genes were all enriched in most of the same pathways of cellular processes, environmental information processing, and organismal systems. However, target genes were enriched in multiple metabolic pathways, whereas genes related to stroke pathology had little effect on metabolic pathways (Figure 4). 61 enriched pathways were shared by all groups. Then, we searched the correlation of the above pathways and stroke in the literature database. The top 6 pathways, including the apoptosis pathway, TNF signaling pathway, VEGF signaling pathway, NF−kappa B signaling pathway, PI3K−Akt signaling pathway, and MAPK signaling pathway, were defined with more than 400 studies. The enriched filtered target genes in these pathways in DTD, XFZYD, and XXMD are also listed (Supplementary Table S8). Integrating these pathways and targets showed that most targets in these pathways were shared by two or three prescriptions, especially in the MAPK signaling pathway and the PI3K/AKT signaling pathway (Figure 5).

### Construction of a Multilevel Network and Key Synergistic Compounds Screening

**FIGURE 6:**
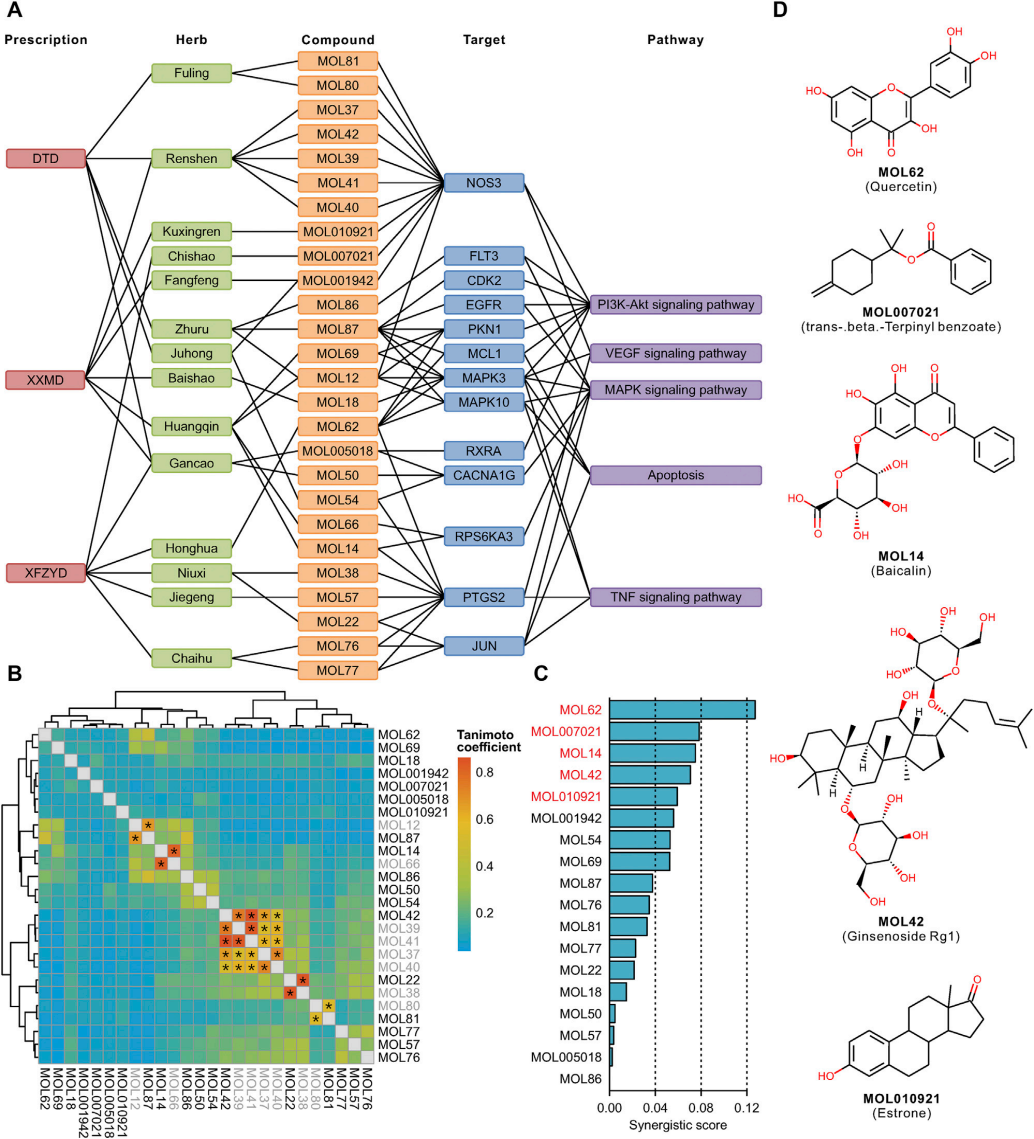
Screening of key compounds by calculating the synergistic score: Network relationship of prescription, herb, compound, target, and pathway **(A)**. Molecular fingerprint similarity of the screened compounds. Compound pairs with similarity >0.5 are marked with an asterisk. Gray color indicates the removed compound **(B)**. Synergistic score of the filtered compounds. The top 5 compounds are shown in red **(C)**. Chemical structure of the top 5 compounds **(D)**.

There were 63 unique filtered targets in the above 6 pathways, and all of their structures were collected from the PDB database. We downloaded these proteins and predicted their potential binding sites (Supplementary Table S9). Molecular docking of the filtered compounds and these target proteins was performed, and the compound-target pairs with high affinity (less than -8 kcal/mol) were screened. Based on these compound-target pairs and information on prescriptions, botanical drugs, and pathways, we constructed a prescription-herb-compound-target-pathway network (Figure 6A). The complex network revealed that the screened targets were involved in multiple pathways, and more than half of the identified compounds targeted NOS3, PTGS2, and MAPK3. After removing the molecular fingerprint similarities higher than 0.5 of these compounds (Figure 6B), the remaining 18 compounds, as key synergistic compounds, were calculated for synergistic scores. Next, the top 5 synergistic scoring compounds were defined as candidate compounds (Figure 6C). These compounds were MOL62 (quercetin), MOL007021 (trans-beta.-terpinyl benzoate), MOL14 (baicalin), MOL42 (ginsenoside Rg1), and MOL010921 (estrone) (Figure 6D).

### Candidate Compounds Synergistically Improve Cell Survival

**FIGURE 7:**
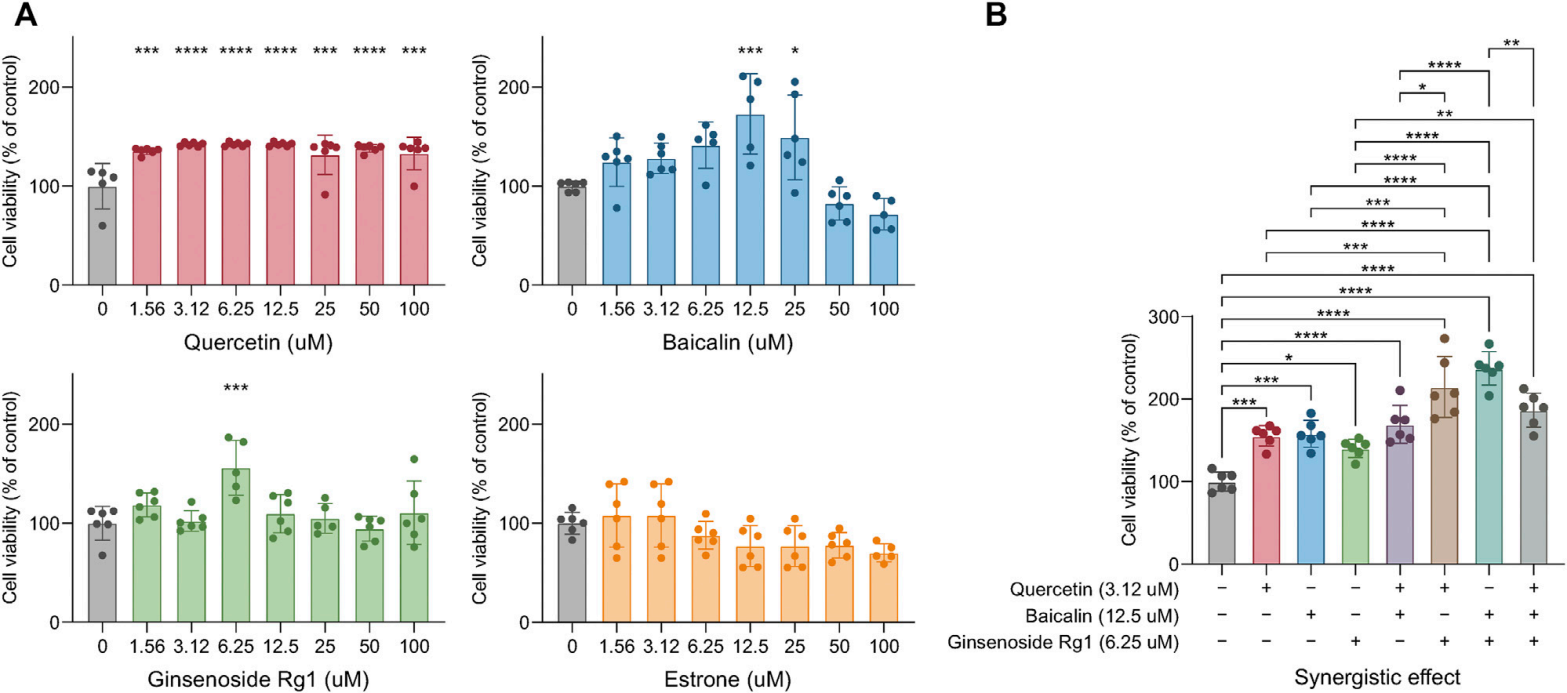
Experimental verification of the compound synergistic effect: The effect of different concentrations of single drugs on cell viability **(A)**. The synergistic effect of three drugs on cell activity **(B)**. Significance: *p < 0.05, **p < 0.01, ***p < 0.001, and ****p < 0.0001.

The OGD/R model was used to verify the reliability of our model, and the independent and synergistic effects of these four compounds on cell viability were deeper explored and analyzed (we did not find a supplier of MOL007021). The results indicated that quercetin significantly improved cell viability from low to high concentrations, compared with the vehicle control group. In addition, baicalin significantly improved cell viability at 12.5 and 25 μM. However, too high of a concentration of baicalin above 50 µM could promote cell death, indicating potential cell cytotoxicity. This was not because the concentration of DMSO as a solvent was too high, even in a 100 uM concentration of Baicalin, DMSO was not more than 1/10,000. Moreover, ginsenoside Rg1 showed activity at only 6.25 μM, and there was no evident cell cytotoxicity in higher concentrations. However, estrone showed no effect on cell viability (Figure 7A). Under the optimal concentrations of these compounds (quercetin: 3.12 μM, baicalin: 12.5 μM, and ginsenoside Rg1: 6.25 μM), the synergetic effect of these three compounds showed better cytoprotective effects in combination, compared with individual drug application. Moreover, the combination of baicalin and ginsenoside Rg1 showed the best protective effect, the combination of quercetin and ginsenoside Rg1 showed the second good effect, whereas the combination of quercetin and baicalin was not so significant (Figure 7B).

### The Binding Mode of the Candidate Compounds and Target Proteins

**FIGURE 8:**
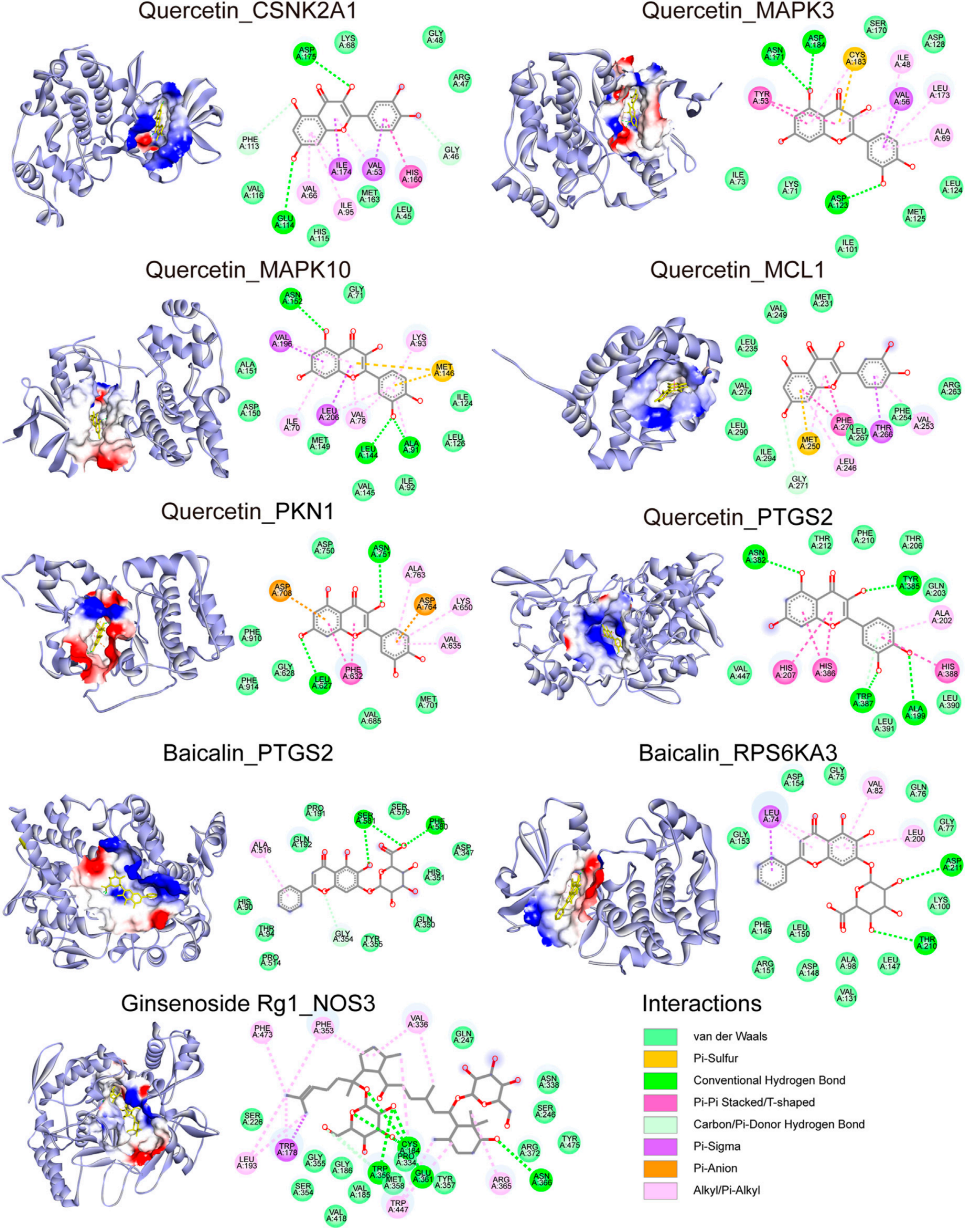
Potential binding mode of the compound and the docking target: The structure of the protein and the docking site of the compound. The bonds between the compounds and the amino acid residues are indicated by colored dashed lines.

The docking modes of the three verified compounds and their targets are shown in Figure 8. Quercetin binds to 6 proteins (CSNK2A1, MAPK3, MAPK10, MCL1, PKN1, and PTGS2). Baicalin binds to 2 proteins (PTGS2 and RPS6KA3); the relevant amino acid residues in PTGS2 are Gly354, Ala516, Phe580, and Ser581, and the relevant amino acid residues in RPS6KA3 are Leu74, Val82, Leu200, Thr210, and Asp211. Ginsenoside Rg1 binds to NOS3 at 11 amino acid residues. Furthermore, the 2D binding pattern diagram shows that quercetin strongly binds to the above proteins, which was consistent with the cell experimental results that demonstrated that quercetin most stably improved cell viability at all concentrations. Furthermore, the targets of ginsenoside Rg1 were complementary with quercetin and baicalin. However, the targets of quercetin and Baicalin overlap, which was also consistent with the synergic effect of the above combinations.

## Discussion

Numerous clinical practices have revealed the significant effect of TCM prescriptions in the treatment of stroke. Combined with modern medical methods, some extracts of several herbs recorded in TCM prescriptions, such as *Calendula officinalis*, *Asarum sieboldii*, *Ginkgo biloba*, and *Salvia miltiorrhiza,* have been widely used in clinical treatment and have shown remarkable effects (Wu et al., 2007; Zhu et al., 2021). Although the use of TCM prescriptions for the treatment of stroke can be traced back to the TCM literature more than 2,000 years ago, and many prescriptions and related herbs are still widely used in the treatment of stroke in China and around the world, there have been insufficient RCTs of TCM prescriptions. Among the TCM prescriptions for the treatment of stroke, only a small number of compound prescriptions provide high-quality evidence through RCTs, such as Neuroaid (Chen et al., 2009; Venketasubramanian et al., 2009; Chen et al., 2013) and Danhong injection (Li B. et al., 2015). There are still many challenges in conducting high-quality RCT research on DTD, XFZYD, and XXMD, such as inconsistent terms of diagnosis and treatment in Chinese medicine, unique theoretical basis, difficulty in designing placebos with the same smell, appearance, and taste, etc. To summarize, although the previous RCTs provided a weak therapeutic recommendation of DTD, XFZYD, and XXMD use on stroke, they still give recommendations for efficacy and safety.

There is still a lack of effective methods to reveal the synergistic effects of multiple TCM prescriptions on complex diseases. To solve this problem, we designed a new model to elucidate the synergistic effect of DTD, XFZYD, and XXMD in stroke treatment by combining pharmacological and chemoinformatic approaches. Then, through molecular docking and network analysis, the key active components in the prescription were determined. Finally, the experimental data was used to verify the reliability of our model. The results showed that quercetin, baicalin, and ginsenoside Rg1 independently and synergistically save damaged neuron cells *in vitro*.

In this study, 28 botanical drugs were checked without HILI report in CNKI and Pubmed databases, and then we found the related hepatotoxicity records in CNKI and TCM-ID databases. Two herbs, including Banxia [Pinellia ternata (Thunb.) Makino (Araceae; Pinellia ternata)] and Nanxing Arisaema heterophyllum Blume (Araceae; Reddish jackinthepulpit), were recorded with potential hepatotoxicity. However, their toxicity were almost eliminated after industry-standard processing with Alunite and Shengjiang [Zingiber officinale Roscoe (Zingiberaceae; fresh ginger)] (Su et al., 2016; Sun et al., 2019; Yu et al., 2019). Although many Chinese medicines are considered to have potential hepatotoxicity, only a small number of herbs have been confirmed to have serious hepatotoxicity. Among the 8,980 traditional Chinese herbs recorded in Chinese pharmacopeia (2015 and 2020) (CommissionNP, 2015; CommissionNP, 2020) and Chinese Materia Medica Dictionary (2006), only 107 herbs have been confirmed to have hepatotoxicity, accounting for 1.2%, such as *Tripterygium wilfordii* (Tian et al., 2019) and *Polygonum multiflorum* (Liu Y. et al., 2019), but in Asia with preference to herbal TCM in China, 11,160 published HILI cases had been identified, all with verified causality by using RUCAM (Roussel Uclaf Causality Assessment Method) (Teschke et al., 2020). Although the liver and kidney toxicity of these herbs has been widely discussed and has caused growing concern, most herbs only have slight, reversible, or dose-related hepatotoxicity. We believe that TCM will be better used in the future with more professional toxic-related studies and clinical evidence accumulation.

Firstly, in the constructed C-T networks of the three prescriptions, the shared compounds accounted for 14.6% of all compounds. Encouragingly, the shared targets accounted for up to 53.7% of all predicted targets. Combined with previous studies, these results suggested that DTD, XFZYD, and XXMD have therapeutic effects with important multitarget drugs and potential synergistic effects (Lapchak, 2011; Kibble et al., 2015). Because of the complex signal network of stroke, intervention and regulation of a single target often cannot change the overall state, and often fail to achieve the expected effect in the treatment of stroke (Casas et al., 2019). The synergy of multitarget drugs in the prescription can improve efficacy and reduce adverse reactions, which has good application prospects (Chou, 2006).

How to eliminate miscellaneous information to identify the synergistic components of multiple prescriptions is a substantial challenge. Our strategy is based on network pharmacology and a conventional collaborative filtering model to recommend synergistic ingredients, thus preliminarily narrowing the scope of the experiment (Ozsoy et al., 2018; Yang M. et al., 2021). In our modified model, the important drugs identified by molecular structure and C-T connectivity tend to have convergent indications. Consequently, the top 50% of the compounds and their targets based on the synergy score were selected for further analysis. The reliability and accuracy of this new method were verified from three aspects: network topology, pathological gene coverage, and functional enrichment coverage. There was a similar network topology, no less than 75% overlap rate of pathological gene intersection, and no less than 79% overlap rate of GO function enrichment. These results showed that the key components filtered out by our model can retain the functionality of the original formula.

Through KEGG enrichment analysis and a literature search, 6 pathways and 63 targets were filtered according to the pathophysiological process of stroke. TNF-α, as an important inflammatory mediator that increases the degree of pathology, causes inflammatory hyperplasia and neuronal apoptosis (Lambertsen et al., 2012; Chen AQ. et al., 2019). In addition, the activation of the VEGF signaling pathway, which produces excessive NO levels, is a key link in brain edema caused by BBB injury (Guix et al., 2005; Casas et al., 2019). Moreover, the NF−κB signaling pathway, the PI3K−Akt signaling pathway, and the MAPK signaling pathway are reportedly involved in the signal transmission of various inflammatory mediators, including the above (Lai et al., 2014). These key genes involved in multiple pathways provide potential therapeutic targets for neuroprotective therapeutic strategies.

After the next strict docking, these potential therapeutic targets and matched compounds were shown in the multilevel network, including 13 remaining targets and 26 compounds. Among these 13 remaining targets, NOS3 and PTGS2 are the targets of most binding compounds. The overactivation of NOS3 after stroke can produce nitric oxide (NO), which enhances the synthesis of proinflammatory mediators, participates in the formation of oxygen free radical damage, and causes brain edema with vascular smooth muscle relaxation (Guix et al., 2005; Casas et al., 2019). PTGS2 metabolizes arachidonic acid released by damaged cells into inflammatory prostaglandins, which recruit inflammatory cells to cause excessive nerve damage (Dore and Shafique Ahmad, 2015). In addition, among these 26 compounds, after removing the molecular fingerprint similarity, 18 compounds were defined as key synergistic compounds. To evaluate the hepatotoxicity, we used three methods, including searching in Pubchem, predicting in ADMT webserver, and Discovery Studio (Supplementary Table S10). The results showed that Baicalin may have slight hepatotoxicity, and isoimperatorin may have serious hepatotoxicity

To verify the reliability of the model, we intend to select the top five compounds in the collaborative ranking for experimental verification. Because trans-beta-terpinyl benzoate was not available for purchase, we verified the independent and synergistic effects of the other four compounds on nerve cell survival with the OGD/R model. Quercetin, baicalin, and ginsenoside Rg1 significantly improved cell viability. Furthermore, the results showed that the synergistic effects of the two compounds were significant, especially the combination of quercetin and ginsenoside Rg1, as well as the combination of baicalin and ginsenoside Rg1, because these two combinations of binding targets were complementary. The OGD/R model simulated neuroinflammation and nerve damage caused by Is/Rep after stroke. Neuroinflammation caused by long-term hypoxia and hemorrhage was more intense, compared with Is/Rep. This was because the contents of the dead cells from long-term hypoxia and the hemoglobin spines after hemorrhagic stroke included more intense inflammatory factors, recruiting a large number of inflammatory cells in a short time and causing more serious neuroinflammation (Rayasam et al., 2018; Hanafy et al., 2019; Tschoe et al., 2020). However, different types of neuroin had similar flammation pathological processes with the same release of inflammatory mediators to aggregate inflammatory cells. For example, overexpressed inflammatory mediators, including matrix metalloproteinase (MMP), high mobility group frame 1 (HMGB1), arachidonic acid metabolites, mitogen-activated protein kinase (MAPK) activation, interleukin-1 (IL-1), and TNF, attracted inflammatory cells, leading to further damage (Jayaraj et al., 2019; Levard et al., 2021). In addition to inflammatory mediators, other common processes, such as the release of toxic excitatory neurotransmitters and damage of the BBB exacerbated more cell death and inflammation (Yang et al., 2019). Thus, our drugs’ effect may be just as effective in other types of neuroinflammation.

Molecular docking showed that Quercetin binds to 6 proteins: CSNK2A1, MAPK3, MAPK10, MCL1, PKN1, and PTGS2; Baicalin binds to 2 proteins: PTGS2 and RPS6KA; Ginsenoside Rg1 binds to NOS3. According to previous studies, Interactions between compounds and targets have been partially confirmed as follows, Quercetin binding resulted in decreased CSNK2A1 protein activity (Wang et al., 2011; Aichinger et al., 2020), influencing phosphorylation of MAPK3 and MAPK10 proteins (Fridrich et al., 2008; Maso et al., 2014; Li Y. et al., 2015; Gupta et al., 2017), promoting MCL1 degradation (Spagnuolo et al., 2011; Russo et al., 2013) and decreasing PTGS2 protein activity (O'Leary et al., 2004; Al-Fayez et al., 2006). Baicalin not only bound to PTGS2 suppressing prostaglandin E2 production but also indirectly decreased PTGS2 expression (Kim et al., 2007; Singh and Chopra, 2014; Park et al., 2017). Ginsenoside Rg1 reportedly inhibited NOS activity (Shen et al., 2007; Chen et al., 2010).

All compounds reportedly relieve neuronal damage through complex and multifaceted mechanisms, including anti-oxidant, anti-inflammatory, anti-excitatory toxicity, and neuronal regeneration-promoting mechanisms *in vitro* and *in vivo*. Previous studies showed that quercetin could decrease the release of TNF-α, IL-1β, and IL-6 by microglia (Zhang et al., 2016), reduce NO and ROS levels (Yang R. et al., 2021), inhibit the destruction of the BBB by MMPs (Lee JK. et al., 2011), and reduce the pro-apoptotic proteins, Bax and caspase-3 (Zhang Y. et al., 2015). Baicalin reportedly decreased protein expression of NF-κB P65, iNOS, COX-2, and MMP9 (Xu et al., 2013; Liang et al., 2017; Chen et al., 2018), protected glutamine synthetase from ROS (Song et al., 2020), and inhibited ferroptosis (Duan et al., 2021). Ginsenosides are the main active components of ginseng, and more than 40 different monomer structures have been identified. Among them, Rg1 has little toxicity, and its neuroprotective effect has been widely studied (Xie et al., 2015). In addition to the similar mechanism of quercetin and baicalin (Zheng et al., 2019), ginsenoside Rg1 reportedly promotes cerebral angiogenesis through the PI3K/Akt/mTOR signaling pathway (Chen J. et al., 2019). Although there have been no clinical trials on stroke treatment, they all have been reported to be safe and have therapeutic effects in the treatment of cardiovascular and skeletal muscle injury repair (Dower et al., 2015; Hang et al., 2018; Bazzucchi et al., 2020; Lee et al., 2021). Based on our criteria, 5 active compounds were finally screened. Preliminary research and our experimental results have shown that quercetin, baicalin, and ginsenoside Rg1 are effective (one compound is unavailable). Due to our strict scoring system, the number of final screened compounds is relatively small. Therefore, more compounds with novel structures may be missed.

According to previous literature, Quercetin (Yang et al., 2015; Zhang Y. et al., 2015; Yang R. et al., 2021), Baicalin (Xu et al., 2013; Lin et al., 2014; Liang et al., 2017; Chen et al., 2018), and Ginsenoside Rg1 (Chen J. et al., 2019) can exert excellent anti-inflammatory and antioxidant effects in the form of monomers. If the monomer compounds taken orally or intragastrically, they will experience similar metabolic pathways in the intestines, which mainly include metabolic processes such as binding to glucose, or binding to glucuronic acid, methylation, and hydrolysis. The main metabolites of Quercetin, Baicalin, and Ginsenoside Rg1 are Quercetin 3-O-β-D-glucuronide (Morand et al., 2000a; b), Baicalein 7-O-glucuronide (Xing et al., 2005; Zhang J. et al., 2015), and M1 (20- O -β- D -glupyranosyl- 20 (S)- protopanaxadiol (Wang et al., 2014; Wang et al., 2016), which are also major conjugates in human plasma, and are distributed throughout the body to exert beneficial functions in target tissues.

In this study, the synergistic effect of TCM compounds was preliminarily explored by combining network pharmacology and chemoinformatic methods. Cellular experiments show that the compounds we screened have a certain synergistic therapeutic effect. This work provides a methodological reference for the optimization and secondary development of TCM prescriptions. Admittedly, there are some limitations to this study. Firstly, the compound and herbal information we collected may not be sufficient. Secondly, the effects of compounds on the human metabolic network are not considered. Thirdly, more filtered key synergistic compounds should be selected for validating the reliability of our model. Finally, the active effect of the synergistic compounds was validated only with *in vitro* assay, without animal assay, and experimental work assessing docking affinity. In future work, we will design a more refined mathematical model to calculate the synergistic effect of the compounds and verify it in animal experiments.

## Data Availability

The original contributions presented in the study are included in the article/Supplementary Material, further inquiries can be directed to the corresponding authors.

## Abbreviations

BBB, blood–brain barrier; C-T, compound-target; DTD, Di-Tan Decoction; NO, nitric oxide; OGD/R, oxygen–glucose deprivation and reoxygenation; TC, Tanimoto coefficient; TCM, Traditional Chinese medicine; TCMSP, Traditional Chinese Medicine Systems Pharmacology; TTD, therapeutic target database; XFZYD, Xue-Fu Zhu-Yu Decoction; XXMD, Xiao-Xu-Ming Decoction.
